# An argumentation semantics for rational human evaluation of arguments

**DOI:** 10.3389/frai.2023.1045663

**Published:** 2023-03-23

**Authors:** Marcos Cramer, Leendert van der Torre

**Affiliations:** ^1^Institute for Artificial Intelligence, TU Dresden, Dresden, Germany; ^2^Department of Computer Science, University of Luxembourg, Esch-sur-Alzette, Luxembourg

**Keywords:** knowledge representation, formal argumentation, abstract argumentation, argumentation semantics, principle-based approach

## Abstract

In abstract argumentation theory, many argumentation semantics have been proposed for evaluating argumentation frameworks. This article is based on the following research question: Which semantics corresponds well to what humans consider a rational judgment on the acceptability of arguments? There are two systematic ways to approach this research question: A normative perspective is provided by the principle-based approach, in which semantics are evaluated based on their satisfaction of various normatively desirable principles. A descriptive perspective is provided by the empirical approach, in which cognitive studies are conducted to determine which semantics best predicts human judgments about arguments. In this article, we combine both approaches to motivate a new argumentation semantics called SCF2. For this purpose, we introduce and motivate two new principles and show that no semantics from the literature satisfies both of them. We define SCF2 and prove that it satisfies both new principles. Furthermore, we discuss findings of a recent empirical cognitive study that provide additional support to SCF2.

## 1. Introduction

The formal study of argumentation is an important field of research within AI (Rahwan and Simari, 2009), in particular in the area of knowledge representation and reasoning, and in the area of multiagent systems. Argumentation as inference provides a general framework for non-monotonic reasoning, and argumentation as dialogue provides a general framework for agent interaction (Prakken, 2018). Argumentation-based approaches are assumed to be better suited for modeling human reasoning than traditional logical methods used in knowledge representation and reasoning, including reasoning in the context of conflicting information and dealing with fallacies and other errors in human reasoning. Formal argumentation is a kind of argument reasoning and is often contrasted with other recent developments in computational argumentation in AI (Van Eemeren and Verheij, 2018), such as approaches based on argument mining (Budzynska and Villata, 2018; Lawrence and Reed, 2020), argument assessment, argument generation, and cognitive modeling (Lauscher et al., 2021).

A central focus of the modern development of formal argumentation has been the idea of Dung (1995) that under some conditions, the acceptance of arguments depends only on a so-called *attack* relation among the arguments, and not on the internal structure of the arguments. Dung called this approach *abstract* argumentation and called the directed graph that represents the arguments and the attack relation between them an *argumentation framework* (*AF*). Whether an argument is deemed acceptable depends on the decision about other arguments. Therefore, the basic concept in abstract argumentation is a *set* of arguments that can be accepted together, called an *extension*. Crucially, there may be several of such extensions, and these extensions may be incompatible. An *extension-based argumentation semantics* takes as input an AF and produces as output a set of extensions.

Traditionally, two classes of extension-based argumentation semantics have been studied (Baroni et al., 2018). Dung introduced several examples of so-called *admissibility-based* semantics, formalizing the idea that an argument is acceptable in the context of an extension if the extension *defends* the argument, i.e., attacks all the attackers of the argument. In this article, we consider his grounded, complete, preferred, and stable semantics. Moreover, we consider the admissibility-based semantics known as semi-stable semantics (Verheij, 1996; Caminada et al., 2012). The other kind of extension-based argumentation semantics is *naive-based* semantics, which is based on the idea that acceptable argument sets are specific maximal conflict-free sets. In this article, we consider the naive, stage, CF2 and stage2 semantics and develop a new naive-based semantics called SCF2. More recently, some semantics have been introduced that are neither admissibility based nor naive based (Dvorák et al., 2022); see the related work section of this article for further details.

Abstract argumentation has various potential applications (Rahwan and Simari, 2009), and the choice of semantics depends on the envisioned application. In this article, we focus on the following research question: Which semantics corresponds well to what humans consider a rational judgment on the acceptability of arguments?

There are two systematic ways to approach this research question: A normative perspective is provided by the *principle-based approach* (Baroni and Giacomin, 2007; van der Torre and Vesic, 2018), in which semantics are evaluated based on their satisfaction of various normatively desirable principles. A descriptive perspective is provided by the *empirical approach* (Rahwan et al., 2010), in which cognitive studies are conducted to determine which semantics best predicts human judgments about arguments. In this article, we combine both approaches.

Two recent empirical cognitive studies on argumentation semantics (Cramer and Guillaume, 2018b, 2019) showed CF2 to be better predictors of human argument evaluation than admissibility-based semantics like grounded and preferred. This finding sheds some doubt on principles that are only satisfied by admissibility-based semantics, e.g., admissibility, defense, and reinstatement, as surveyed by van der Torre and Vesic (2018). For this reason, in this article, we focus on other existing principles (e.g., directionality) and introduce new ones.

The first new principle we consider is *irrelevance of Necessarily Rejected Arguments* (*INRAs*). Informally, INRA says that if an argument is attacked by every extension of an AF, then deleting this argument should not change the set of extensions. The idea, here, is that an argument that is attacked by every extension would be rejected by any party in a debate and hence would never be brought up in a debate. Hence, it should be treated as if it did not even exist.

The second principle that we consider is *Strong Completeness Outside Odd Cycles* (*SCOOCs*). Informally, SCOOC says that if an argument *a* and its attackers are not in an odd cycle, then an extension not containing any of *a*'s attackers must contain *a*. The principle is based on the idea that it is generally desirable that an argument that is not attacked by any argument in a given extension should itself be in that extension. While it is possible to ensure this property in AFs without odd cycles, this is not the case for AFs involving an odd cycle. The idea behind the SCOOC principle is to still satisfy this property as much as possible, i.e., whenever the argument under consideration and its attackers are not in an odd cycle.

We show that of the nine common semantics mentioned earlier, the only ones that satisfy INRA are grounded, complete and naive semantics. In addition, we show that a variant of CF2 that we call nsa(CF2) and that consists of first deleting all self-attacking arguments and then applying CF2 semantics also satisfies INRA.

Furthermore, we show that of these 10 semantics (the nine mentioned at the beginning and nsa(CF2)), the only one that satisfies SCOOC is the stable semantics. However, stable semantics satisfies neither directionality nor INRA. The fact that none of the considered existing semantics satisfies both new principles introduced in this article raises the question whether these two principles can be satisfied in conjunction. We answer this question positively by defining a novel semantics called *SCF2 semantics* that satisfies both of them.

Finally, we discuss the findings of a recent cognitive study by Cramer and Guillaume (2019) and observe that SCF2 explains the judgments of participants in this study better than any existing semantics. This provides additional support for our claim that SCF2 corresponds well to what humans consider a rational judgment on the acceptability of arguments.

This article is an extended version of a workshop article (Cramer and van der Torre, 2019). Compared to the workshop article, here, we give more background on the relation to human-centric AI and consider much more principles from the abstract argumentation literature: While in the workshop article, we focused on three principles, this article evaluates the new semantics against 37 principles. Furthermore, unlike in the workshop article, we give full proofs for all theorems that we present.

### 1.1. Relation to human-centric intelligence

Humans use arguments both as a means to persuade others in a dialogue and as a way to make decisions and draw tentative conclusions by comparing arguments for and against various positions. In order for AI technology to interact meaningfully with humans, argumentation as practiced by humans, therefore, needs to be taken into account.

Argumentation and dialogue have been studied in many fields. In artificial intelligence, a distinction can be made between formal argumentation and computational argumentation, where formal argumentation is concerned both with argumentation as inference studied in knowledge representation and reasoning and argumentation as dialogue studied in multiagent systems (Prakken, 2018). Since the work of Dung (1995), these approaches are studied not only at a logical or structured level but also at an abstract level.

Formal argumentation can be seen as a natural successor of logic-based approaches studied in the previous century (Prakken, 2018; Van Eemeren and Verheij, 2018). Approaches to human reasoning based on classical logic have little to say in case of conflict. However, humans need to reason about conflict all the time, for example, when receiving contradictory or false information or when dealing with opposing opinions. Formal argumentation goes beyond classical logic by presenting distinct rational viewpoints in case of conflict and by incorporating methods from non-monotonic logic to resolve some of these conflicts. They do this by modeling facts as assumptions and modeling rules as defeasible inferences. On the other hand, formal argumentation builds on traditional logical methods by representing the structure of individual arguments themselves in a logical way. Each extension of a set of acceptable arguments may be seen as a coherent viewpoint.

In the current article, the focus is on argumentation as inference and on abstract argumentation, the study of the relation among arguments with a focus on how the attack relation between arguments (when one argument is a counterargument to another) can serve as a basis for judgments about the acceptability of arguments. It can be seen as the study of a dialogue state at a single moment in time. Even when an argument is not accepted in any extension and thus can be ignored according to the INRA principle, the same argument can play a role later in the dialogue when the framework has changed.

Dung's theory is based on the assumption that the acceptance of arguments depends only on the attack relation among the constructive arguments, not on their internal structure. Dung's theory can be defended in different ways. Suppose the assumption is false, i.e., one of the dialogue participants believes that due to the internal structure of argument *A*, it cannot be accepted. Now suppose that another dialogue participant disagrees with this position and claims that the internal structure of the argument is completely fine. In this disagreement, we can model this disagreement with arguments *B* and *C* and the relation between arguments *A* and *B* with an attack from *B* to *A*. In general, the fact that in abstract argumentation, everything has to be modeled by an argument can be interpreted as the statement that every criticism can be criticized itself as well.

The methods of abstract argumentation are also relevant for the study of the internal structure of arguments and the dynamics of dialogue scenarios. When the internal structure of arguments is made explicit, and the arguments are attributed to the agents that put them forward, one can address how arguments are generated in light of other arguments and how that can lead to a resolution of conflicts and paradoxes. In such cases, the argumentation framework can change over time due to agent interaction.

Human reasoning is inherently non-monotonic: It often happens that one draws a conclusion from certain given information but later gives up that conclusion due to novel information speaking against it. This non-monotonicity of human reasoning cannot be modeled in classical monotonic logic. For this reason, non-monotonic logic has been designed since the 1980s. Since its inception in the early 1990s, formal argumentation has had a strong connection to non-monotonic logic. The idea, here, is that novel information allows us to construct new arguments, some of which may attack previously accepted arguments and lead to their rejection. Thus, formal argumentation can often be viewed as a tool for making the inference process of non-monotonic logics explicit, concrete, and close in nature to actual human reasoning.

While some of the research in formal argumentation is somewhat detached from the human practice of argumentation, there are also many researchers who aim at building a bridge between human reasoning and formal argumentation by studying how various formalisms and semantics from formal argumentation relate to actual human reasoning. For example, formal argumentation has been combined with approaches based on natural language processing and argument mining (Budzynska and Villata, 2018). Furthermore, as detailed in Section 6, multiple cognitive studies have been conducted to investigate the relation between human reasoning and argumentation formalisms.

With the help of such interdisciplinary research, formal argumentation is becoming more relevant to the endeavor of human-centric AI. This article aims to contribute to this research by studying which argumentation semantics (i.e., which method for evaluating the acceptability of arguments based on the attack relation between the arguments) is a good model for rational human evaluation of arguments. For this, two approaches are combined as follows:

- A normative perspective is provided by the principle-based approach, in which semantics are evaluated based on their satisfaction of various normatively desirable principles.
- A descriptive perspective is provided by the empirical approach, in which cognitive studies are conducted to determine which semantics best predicts human judgments about arguments.

In this article, we argue that the SCF2 semantics is a reasonable choice from both points of view. It may thus be better suited for human-centric AI than other argumentation semantics proposed in the literature.

## 2. Preliminaries

In this section, we define required notions from abstract argumentation theory Dung (1995) and Baroni et al. (2018). In addition, we define three principles from the literature on principle-based argumentation (Baroni and Giacomin, 2007; van der Torre and Vesic, 2018) and present an argument for the case that the directionality principle is a desirable property for a semantics designed to match what humans would consider a rational judgment on the acceptability of arguments.

DEFINITION 1. An *argumentation framework (AF)* *F* = 〈*Ar, att*〉 is a finite directed graph in which the set *Ar* of vertices is considered to represent arguments and the set *att* of edges is considered to represent the attack relation between arguments, i.e., the relation between a counterargument and the argument that it counters.

DEFINITION 2. An *att*-path is a sequence 〈*a*_0_, …, *a*_*n*_〉 of arguments where (*a*_*i*_, *a*_*i*+1_) ∈ *att* for 0 ≤ *i* < *n* and where *a*_*j*_ ≠ *a*_*k*_ for 0 ≤ *j* < *k* ≤ *n* with either *j* ≠ 0 or *k* ≠ *n*. An *odd* *att-cycle* is an *att*-path 〈*a*_0_, …, *a*_*n*_〉 where *a*_0_ = *a*_*n*_ and *n* is odd.

DEFINITION 3. Let *F* = 〈*Ar, att*〉 be an AF, and let *S* ⊆ *Ar*. We write *F*|_*S*_ for the restricted AF 〈*S, att* ∩ (*S* × *S*)〉. The set *S* is called *conflict-free* iff there are no arguments *b, c* ∈ *S* such that *b* attacks *c* (i.e., such that (*b, c*) ∈ *att*). Argument *a* ∈ *Ar* is *defended* by *S* iff for every *b* ∈ *Ar* such that *b* attacks *a* there exists *c* ∈ *S* such that *c* attacks *b*. We say that *S* *attacks* *a* if there exists *b* ∈ *S* such that *b* attacks *a*, and we define *S*^+^ = {*a* ∈ *Ar* ∣ *S*attacks*a*} and *S*^−^ = {*a* ∈ *Ar* ∣ *a* attacks some *b* ∈ *S*}.

- *S* is a *complete extension* of *F* iff it is conflict free, it defends all its arguments, and it contains all the arguments it defends.
- *S* is a *stable extension* of *F* iff it is conflict free, and it attacks all the arguments of *A* \ *S*.
- *S* is the *grounded extension* of *F* iff it is a minimal with respect to set inclusion complete extension of *F*.
- *S* is a *preferred extension* of *F* iff it is a maximal with respect to set inclusion complete extension of *F*.
- *S* is a *semi-stable extension* of *F* iff it is a complete extension, and there exists no complete extension *S*_1_ such that $S\cup S^{+}\subset S_{1}\cup S_{1}^{+}$.
- *S* is a *stage extension* of *F* iff *S* is a conflict-free set, and there exists no conflict-free set *S*_1_ such that $S\cup S^{+}\subset S_{1}\cup S_{1}^{+}$.
- *S* is a *naive extension* of *F* iff *S* is a maximal conflict-free set.

CF2 semantics was first introduced by Baroni et al. (2005). The idea behind it is that we partition the AF into *strongly connected components* and recursively evaluate it component by component by choosing maximal conflict-free sets in each component and removing arguments attacked by chosen arguments. We formally define it following the notation of Dvořák and Gaggl (2016). For this, we first need some auxiliary notions:

DEFINITION 4. Let *F* = 〈*Ar, att*〉 be an AF, and let *a, b* ∈ *Ar*. We define *a* ~ *b* iff either *a* = *b* or there is an *att*-path from *a* to *b*, and there is an *att*-path from *b* to *a*. The equivalence classes under the equivalence relation ~ are called *strongly connected components* (SCCs) of *F*. We denote the set of SCCs of *F* by *SCCs*(*F*). Given *S* ⊆ *Ar*, we define *D*_*F*_(*S*): = {*b* ∈ *Ar* ∣ ∃*a* ∈ *S*:(*a, b*) ∈ *att* ∧ *a* ≁ *b*}.

If *F* = 〈∅, ∅〉, we consider ∅ to be an SCC of *F*; else ∅ is not an SCC.

The simplified SCC-recursive scheme used for defining CF2 and stage2 is a function that maps a semantics σ to another semantics scc(σ):

DEFINITION 5. Let σ be an argumentation semantics. The argumentation semantics scc(σ) is defined as follows. Let *F* = 〈*Ar, att*〉 be an AF, and let *S* ⊆ *Ar*. Then *S* is an scc(σ)-extension of *F* iff either

- |*SCCs*(*F*)| ≤ 1 and *S* is a σ-extension of *F*, or
- |*SCCs*(*F*)| > 1 and for each *C* ∈ *SCCs*(*F*), *S* ∩ *C* is an scc(σ)-extension of *F*|_*C*\_*D*__*F*_(*S*)_.

*CF2 semantics* is defined to be scc(*naive*), and *stage2* semantics is defined to be scc(*stage*).

Apart from the function scc, we introduce a further function—called nsa—that also maps a semantics to another semantics. Informally, the idea behind nsa(σ) is that we first delete all self-attacking arguments and then apply σ. To define nsa formally, we first need an auxiliary definition:

DEFINITION 6. Let *F* = 〈*Ar, att*〉 be an AF. We define the *non-self-attacking restriction* of *F*, denoted by *NSA*(*F*), to be the AF $F_{{\text{Ar}}^{\prime }}$, where *Ar*′: = {*a* ∈ *Ar* ∣ (*a, a*)∉*att*}.

DEFINITION 7. Let σ be an argumentation semantics. The argumentation semantics nsa(σ) is defined as follows. Let *F* = 〈*Ar, att*〉 be an AF, and let *S* ⊆ *Ar*. We say that *E* is an nsa(σ)-extension of *F* iff *E* is a σ-extension of *NSA*(*F*).

We now define the directionality principle introduced by Baroni and Giacomin (2007). For this, we first need an auxiliary notion:

DEFINITION 8. Let *F* = 〈*Ar, att*〉 be an AF. A set *U* ⊆ *Ar* is *unattacked* iff there exists no *a* ∈ *A* \ *U* such that *a* attacks some *b* ∈ *U*.

DEFINITION 9. A semantics σ satisfies the *directionality* principle iff for every AF *F* and every unattacked set *U*; it holds that σ(*F*|_*U*_) = {*E* ∩ *U* ∣ *E* ∈ σ(*F*)}.

The directionality principle corresponds to an important feature of the human practice of argumentation, namely that if a person has formed an opinion on some arguments and is confronted with new arguments, they will only feel compelled to reconsider their judgment on the prior arguments if one of the new arguments attacks one of the prior arguments. Apart from our own intuition, we can also refer to the results of an empirical cognitive study on argumentation that shows that humans are able to systematically judge the directionality of attacks between arguments (Cramer and Guillaume, 2018a). Thus, we consider the directionality principle crucial for the goal that we focus on in this article.

We define two further principles from the literature on principle-based argumentation (Baroni and Giacomin, 2007; van der Torre and Vesic, 2018) that are relevant for getting a better picture of the behavior of a semantics and can be used to derive multiple further principles proposed in the literature.

DEFINITION 10. A semantics σ satisfies the *naivety* principle if and only if for every AF *F*, for every *E* ∈ σ(*F*), *E* is a maximal with respect to set inclusion conflict-free set in *F*.

DEFINITION 11. Given an argumentation framework *F* = (*Ar, att*) and sets *S, E* ⊆ *Ar*, we define *U*_*F*_(*S, E*): = {*a* ∈ *S* ∣ ∄*b* :(*b, a*) ∈ *att, b* ≁ *a*, and *E* does not attack *b*}.

DEFINITION 12. A binary function *BF* is called a *base function* iff for every AF *F* = (*Ar, att*) such that |*SCCs*(*F*)| = 1 and every set *C* ⊆ *Ar*, BF(F,C)⊆P(Ar).

Here the notation P(Ar) denotes the powerset of *Ar*, i.e., the set of all subsets of *Ar*.

DEFINITION 13. Given a base function *BF*, an AF *F* = (*Ar, att*) and a set *C* ⊆ *Ar*, we recursively define GF(BF,F,C)⊆P(Ar) as follows: for every *E* ⊆ *Ar*, *E* ∈ *GF*(*BF, F, C*) iff

- in case |*SCCs*(*F*)| = 1, *E* ∈ *BF*(*F, C*),
- otherwise, for all *S* ∈ *SCCs*(*F*), (*E* ∩ *S*) ∈ *GF*(*BF*, *F*|_*S*\_*D*__*F*_(*E*)_, *U*_*F*_(*S, E*) ∩ *C*).

DEFINITION 14. A semantics σ satisfies the *SCC-recursiveness* principle iff there is a base function *BF* such that for every AF *F* = (*Ar, att*) we have σ(*F*) = *GF*(*BF, F, Ar*).

## 3. Two new principles

In this section, we define and motivate the two new principles introduced in the article. Let us first look at the principle that we call *Irrelevance of Necessarily Rejected Arguments* (*INRAs*). The idea behind this principle is that in order for an argument to be relevant in a debate, there must be a coherent standpoint according to which this argument is accepted or at least not clearly rejected. If an argument is attacked by an extension, it would be clearly rejected by any rational person whose standpoint is described by the extension in question. So, if an argument is attacked by every extension, it is clearly rejected in light of every rational standpoint and would, therefore, never be brought up in a debate between rational people. For the purpose of evaluating the acceptability of arguments, it, therefore, makes sense to treat such an argument as if it did not even exist. Talking in the language of extensions, this can be formulated as follows: If an argument *a* is attacked by every extension of an AF, then deleting *a* should not change the set of extensions.^1^

In order to formally define the INRA principle, we first need to define a notation for an AF with one argument deleted:

DEFINITION 15. Let *F* = 〈*Ar, att*〉 be an AF and let *a* ∈ *Ar* be an argument. Then *F*^−*a*^ denotes the restricted AF *F*|_*Ar*\{*a*}_.

DEFINITION 16. Let σ be an argumentation semantics. We say that σ satisfies *Irrelevance of Necessarily Rejected Arguments* (*INRA*) iff for every AF *F* = 〈*Ar, att*〉 and every argument *a* ∈ *Ar*, if every *E* ∈ σ(*F*) attacks *a*, then σ(*F*) = σ(*F*^−*a*^).

**Figure 1:**
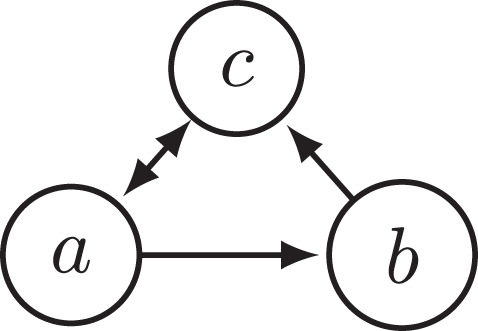
Argumentation framework *F*_1_.

We now illustrate the definition through an example of the preferred semantics:

EXAMPLE 1. Consider the argumentation framework *F*_1_ depicted in Figure 1. The only preferred extension of *F*_1_ is {*a*}. This extension attacks *b*. So, *b* is attacked by every extension of *F*_1_. If we remove argument *b* from *F*_0_, we are left with the AF $F_{1}^{-b}$ consisting only of *a* and *c* attacking each other. $F_{1}^{-b}$ has two preferred extensions, {*a*} and {*c*}. So, when removing an argument (namely *b*) that was attacked by every extension, the set of extensions changed. Thus, this example constitutes a violation of the INRA principle. We have, therefore, established that the preferred semantics does not satisfy INRA.

The second principle that we consider is *Strong Completeness Outside Odd Cycles* (*SCOOC*). Informally, SCOOC says that if an argument *a* and its attackers are not in an odd cycle, then an extension not containing any of *a*'s attackers must contain *a*.

In order to formally define the Strong Completeness Outside Odd Cycles principle, we first need to define a notation for the set of all attackers of an argument and the auxiliary notion of a set of arguments being *strongly complete outside odd cycles*.

DEFINITION 17. Let *F* = 〈*Ar, att*〉 be an AF, and let *A* ⊆ *Ar*. We say that *A* is *strongly complete outside odd cycles* iff for every argument *a* ∈ *Ar*, the following condition holds: If

- no argument in {*a*}∪{*a*}^−^ is in an odd *att*-cycle, and
- *A* ∩ {*a*}^−^ = ∅,

then *a* ∈ *A*.

DEFINITION 18. Let σ be an argumentation semantics. We say that σ satisfies *Strong Completeness Outside Odd Cycles (SCOOC)* iff for any AF *F*, every σ-extension of *F* is strongly complete outside odd cycles.

Before motivating the SCOOC principle, we first illustrate it with an example of a violation of the principle in the CF2 semantics.

**Figure 2:**
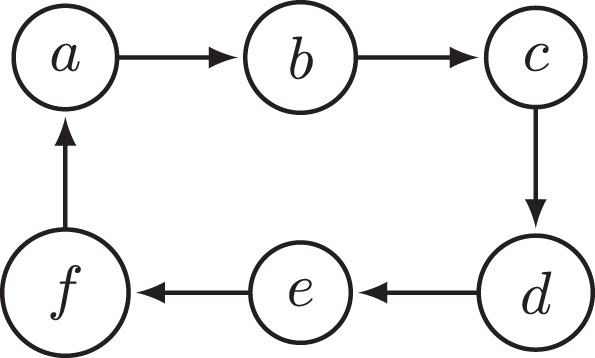
Argumentation framework *F*_2_.

EXAMPLE 2. Consider the argumentation framework *F*_2_ depicted in Figure 2. It is a simple six-cycle. One of the CF2 extensions of *F*_2_ is *E* = {*a, d*}. *F*_2_ contains no odd cycle, so in particular *b* and *c* are not in an odd cycle. Since {*c*}^−^ = {*b*}, this means that no argument in {*c*}∪{*c*}^−^ is in an odd cycle. Moreover, *E* ∩ {*c*}^−^ = ∅. Thus, for *E* to be strongly complete outside odd cycles, it would have to contain *c*. However, *c* ∉ *E*, so *E* is not strongly complete outside odd cycles. We have, therefore, established that the CF2 semantics does not satisfy SCOOC.

The SCOOC principle is related to the property of *strong completeness*: An extension *E* is *strongly complete* iff every argument not attacked by *E* is in *E*. We call this property *strong completeness* as it is a strengthening of completeness, which states that every argument defended by *E* is in *E*.

The stable semantics is the only widely studied argumentation semantics that satisfies strong completeness. More precisely, the stable semantics can be characterized by the conjunction of conflict freeness and strong completeness. In other words, one can say that the stable semantics is motivated by the idea that a violation of strong completeness constitutes a paradox and should therefore be avoided.

The stable semantics satisfies strong completeness at the price of allowing for situations in which there are no extensions, and hence no judgment can be made on any argument whatsoever. Such cases are always due to odd *att*-cycles. So, we can say that odd *att*-cycles—unless resolved through arguments attacking the odd cycle—cause paradoxical situations. The idea of most semantics other than stable semantics is to somehow contain these paradoxes, so that they do not affect our ability to make judgments about completely or sufficiently unrelated arguments.

The idea of the SCOOC principle is that while in odd cycles we may not be able to avoid paradoxical judgments about the arguments, i.e., a judgment in which an argument is not accepted even though none of its attackers is accepted, such paradoxical judgments should be completely avoided outside of odd cycles.

How does that differ from the containment of paradoxical situations provided by existing semantics? Admissibility-based semantics do not allow for any judgment about an argument in an unattacked odd cycle; however, this undecided status is not limited to odd cycles but carries forward to arguments that are not in an odd cycle but that are *att*-reachable from an odd cycle.

Naive-based semantics like CF2, stage, and stage2 allow for judgments about arguments in an unattacked odd cycle but also at the cost of affecting the way arguments that are not in odd cycles are interpreted. For example, as established in Example 2 earlier, CF2 allows for a six-cycle to be interpreted in a doubly paradoxical way despite the fact that it is an even cycle that can be interpreted in a non-paradoxical manner. This behavior of CF2 was also considered problematic by Dvořák and Gaggl (2016), who used this example to motivate their stage2 semantics, but as we will show in **Figure 6**, stage2 also fails to avoid paradoxical judgments about arguments that are not themselves involved in an odd cycle.

The SCOOC principle was designed to systematically identify whether a semantics suffers from this problem. As it turns out, all the standard semantics other than stable do suffer from the problem, i.e., do not satisfy SCOOC.

We will now look at which semantics satisfy or do not satisfy each of the two principles that we have defined.

THEOREM 1. The grounded, complete, naive, and nsa(CF2) semantics satisfy INRA.

Before we can prove the theorem, we first need some auxiliary definitions and lemmas.

DEFINITION 19. A semantics σ is called *SCC-rich* iff for every AF *F* = 〈*Ar, att*〉 such that |*SCCs*(*F*)| = 1 and every argument *a* ∈ *Ar*, there is an extension *E* ∈ σ(*F*) such that *E* does not attack *a*.

DEFINITION 20. A semantics is called *semi-rich* iff for every AF *F* = 〈*Ar, att*〉 and every argument *a* ∈ *Ar* such that (*a, a*)∉*att*, there is an extension *E* ∈ σ(*F*) such that *E* does not attack *a*.

DEFINITION 21. A semantics is called *SCC-semi-rich* iff for every AF *F* = 〈*Ar, att*〉 such that |*SCCs*(*F*)| = 1 and every argument *a* ∈ *Ar* such that (*a, a*)∉*att*, there is an extension *E* ∈ σ(*F*) such that *E* does not attack *a*.

LEMMA 1. Naive semantics is semi-rich and thus also SCC-semi-rich.

PROOF. Let *F* = 〈*Ar, att*〉 be an AF and let *a* ∈ *Ar* be an argument such that (*a, a*)∉*att*. Let *E* be a naive extension of $F{|}_{\text{Ar}\backslash (\left\{a\right\}\cup {\left\{a\right\}}^{+}\cup {\left\{a\right\}}^{-}}$. Then, *E*∪{*a*} is a naive extension of *F* and *E*∪{*a*} does not attack *a*.     □

LEMMA 2. Grounded and complete semantics are SCC-rich.

PROOF. Let *F* = 〈*Ar, att*〉 be an AF such that |*SCCs*(*F*)| = 1 and let *a* ∈ *Ar*. We distinguish two cases:

1. *att* = ∅. In this case, *Ar* is the only grounded and complete extension of *F*, and *Ar* does not attack *a*.
2. *att* ≠ ∅. Since |*SCCs*(*F*)| = 1, this implies that every argument is attacked by some argument. Thus ∅ is a grounded and complete extension of *F*. Since ∅ does not attack *a*, the required condition is satisfied.

     □

The following lemma has a very technical proof that we provide in Appendix 1. Here, we just sketch the main idea of the proof and then discuss what is the main difficulty in making the argument rigorous.

LEMMA 3. Let σ be an SCC-rich or SCC-semi-rich semantics.

1. If σ is SCC-rich, then scc(σ) satisfies INRA.
2. If σ is SCC-semi-rich, then nsa(scc(σ)) satisfies INRA.

PROOF SKETCH. First, we observe that for showing that nsa(scc(σ)) satisfies INRA, it is enough to consider AFs without self-attacking arguments. However, in such AFs, SCC-richness, and SCC-semi-richness coincide. So, we can actually assume SCC-richness for both parts of the lemma.

We consider an argument *a* that is attacked by every extension and need to show that removing that argument from the AF will not result in the emergence of new extensions or the disappearance of any previous extensions. Due to the SCC-richness of σ, *a* cannot be in an initial SCC. Instead, *a* must be in a position where, whatever happens in the SCCs that come before *a*, some argument attacking *a* will be accepted. Thus, the SCC-recursive scheme removes *a* from the computation of the semantics at that step. Since that is the case, removing *a* from the AF will make no difference because what happens in the SCCs that preceded *a* will not be affected by the initial removal of *a*, and starting at the SCC that (originally) contains *a*, it makes no difference whether *a* is initially removed from the framework or removed from the computation by the SCC-recursive scheme due to having an attacker from a previous SCC.

     □

The main difficulty in making this proof sketch a rigorous proof is that the removal of *a* may change the structure of the SCCs, as the SCC containing *a* may be split up into multiple SCCs. That complicates the argument significantly, but the rigorous proof in Appendix 1 spells out in detail how the argument works to cover this case.

PROOF OF THEOREM 1. By Lemmas 1, 2, and 3 and the fact that grounded = scc(grounded), complete = scc(complete), and nsa(CF2) = nsa(scc(naive)), it directly follows that grounded, complete and nsa(CF2) satisfy INRA.

We now show that naive semantics satisfies INRA. Let *F* = 〈*Ar, att*〉 be an AF and let *a* ∈ *Ar* be an argument such that for every *E* ∈ naive(*F*), *E* attacks *a*. By the semi-richness of the naive semantics (Lemma 1), it follows that (*a, a*) ∈ *att*.

We need to show that naive(*F*) = naive(*F*^−*a*^). Let *S* ∈ naive(*F*). As *a* ∉ *S*, *S* ⊆ *Ar*\{*a*}. *S* is conflict free, and as *S* is maximal with this property in *F*, it is also maximal with this property in *F*^−*a*^. So *S* ∈ naive(*F*^−*a*^), as required.

Now, let *S* ∈ naive(*F*^−*a*^). *S* is conflict free. Since (*a, a*) ∈ *att*, *S*∪{*a*} is not conflict free. Together with the maximality of *S* in *F*^−*a*^, this implies that *S* is a maximally conflict free subset of *Ar*, i.e., *S* ∈ naive(*F*), as required.     □

THEOREM 2. Stable, preferred, semi-stable, stage, stage2, and CF2 semantics violate INRA.

**Figure 3:**
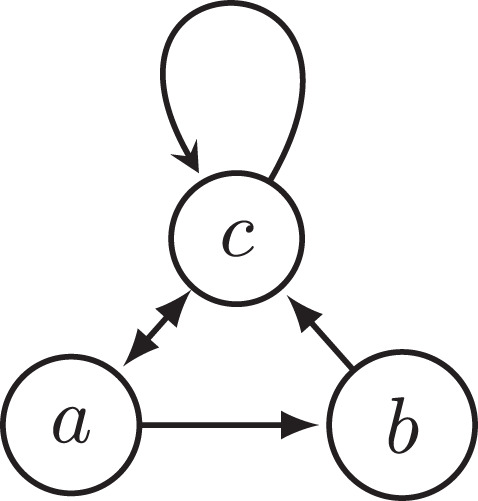
Argumentation framework *F*_3_. It shows that CF2 semantics violates INRA since both extensions ({*a*} and {*b*}) attack *c*, but after removing *c*, {*b*} is no longer an extension.

PROOF. The fact that the preferred semantics violates INRA was already established in Example 1 with reference to the argumentation framework *F*_1_. The same argumentation framework also constitutes a violation of INRA for the stable, semi-stable, stage, and stage2 semantics, as these semantics coincide with the preferred semantics on *F*_1_ and $F_{1}^{-b}$. A counterexample of CF2 semantics is shown in Figure 3, as explained in the caption of the figure.

THEOREM 3. Stable semantics satisfies SCOOC.

PROOF. Consider an AF *F*, a stable extension *E* of *F* and an argument *a* ∈ *Ar*, such that *E* ∩ {*a*}^−^ = ∅. Then, by definition of stable semantics, we have *a* ∈ *E*. Consequently, *E* is strongly complete, and in particular, *E* is strongly complete outside odd cycles.

THEOREM 4. Complete, grounded, preferred, semi-stable, naive, stage, CF2, stage2, and nsa(CF2) semantics violate SCOOC.

PROOF. The counterexample of CF2 was already presented in Example 2. The argumentation framework *F*_2_ from that example (the simple six-cycle) also constitutes a counterexample of naive and nsa(CF2), as they agree with CF2 on the simple six-cycle.

**Figure 4:**
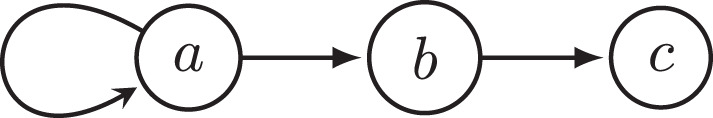
Argumentation framework *F*_4_. It shows that complete, grounded, preferred, and semi-stable semantics violate SCOOC since *E* = {} is an extension, but *E* is not strongly complete outside odd cycles: *b* and *c* are not in an odd cycle, {*c*}^−^ = {*b*}, but *E* does not contain *c*.

**Figure 5:**
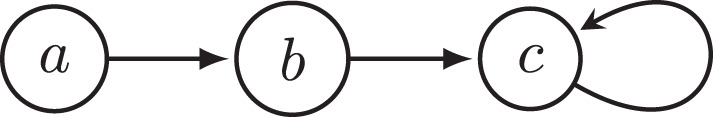
Argumentation framework *F*_5_. It shows that stage and naive semantics violate SCOOC since *E* = {*b*} is an extension, but *E* is not strongly complete outside odd cycles: *a* is not in an odd cycle, {*a*}^−^ = {}, but *E* does not contain *a*.

**Figure 6:**
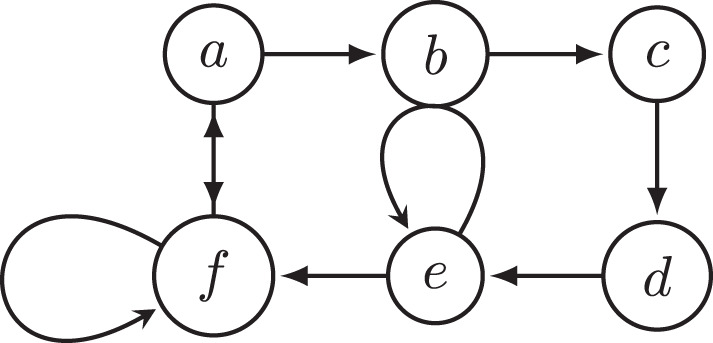
Argumentation framework *F*_6_. It shows that stage2 semantics violates SCOOC since *E* = {*a, d*} is an extension, but *E* is not strongly complete outside odd cycles: *b* and *c* are not in an odd cycle, {*c*}^−^ = {*b*}, but *E* does not contain *c*.

A counterexample of complete, grounded, preferred, and semi-stable is shown in Figure 4, and a counterexample of naive and stage is shown in Figure 5, and a counterexample of stage2 is shown in Figure 6.

Note that for a framework that does not contain any odd cycles at all, the preferred, and semi-stable extensions coincide with the stable extensions, so that in this special case, the SCOOC principle is also satisfied for the preferred and semi-stable semantics.

## 4. SCF2 semantics

In this section, we define and study the new semantics SCF2, which satisfies both of the new principles introduced in the previous section and the three principles defined in the preliminaries. Furthermore, we will motivate the design choices in the definition of SCF2 by looking at how semantics defined in a similar way as SCF2 fail to satisfy at least one of directionality, INRA or SCOOC.

### 4.1. Definition of SCF2 and examples

We have seen in the previous section that nsa(CF2) satisfies INRA but does not satisfy SCOOC. The idea behind the definition of SCF2 is that we modify the definition of nsa(CF2) by already enforcing SCOOC at the level of the single SCCs considered in the SCC-recursive definition of nsa(CF2). For this, we define a variant of naive semantics called *SCOOC-naive semantics*.

DEFINITION 22. Let *F* = 〈*Ar, att*〉 be an AF, and let *A* ⊆ *Ar*. We say that *A* is an *SCOOC-naive extension* of *F* if *A* is subset-maximal among the conflict-free subsets of *Ar* that are strongly complete outside odd cycles.

Recall that CF2 is defined to be scc(naive), i.e., nsa(CF2) = nsa(scc(naive)). To define SCF2, we just replace naive semantics by SCOOC-naive semantics in this definition.

DEFINITION 23. SCF2 semantics is defined to be nsa(scc(SCOOC-naive)).

In other words, SCF2 works by first deleting all self-attacking arguments and then applying the SCC-recursive scheme that is also used in the definition of CF2, but applying SCOOC-naive semantics instead of naive semantics to each single SCC.

The computation of the SCF2 extensions of a given argumentation framework *F* can be described through the following non-deterministic algorithm:

1. Delete all self-attacking arguments from *F*.
2. Assign *E*: = ∅.
3. Divide *F* into strongly connected components (SCCs).
4. Choose some initial SCC *C* of *F*.
5. Choose a maximal conflict-free subset *A* of *C* that satisfies the SCOOC principle.
6. Assign *E*: = *E* ∪ *A*.
7. Delete all arguments in *C* and all arguments attacked by *A* from *F*.
8. If *F* still contains arguments, go to step 3.
9. Return *E*.

**Figure 7:**
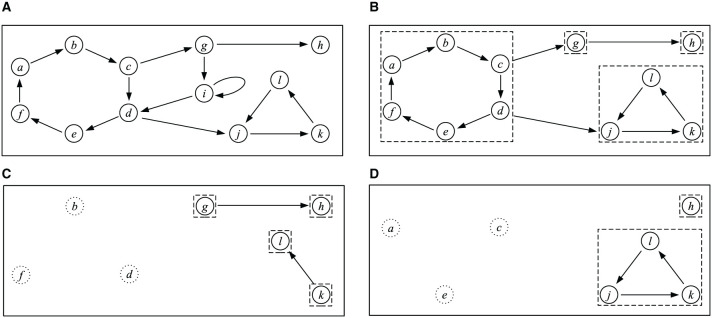
**(A)** Argumentation framework *F*_7_. **(B–D)** Intermediary steps in the computation of the SCF2 extensions of *F*_7_. Dashed lines indicate SCCs. Arguments in dotted circles have already been chosen to be included in the extension and are no longer part of the AF under consideration.

EXAMPLE 3. Consider the argumentation framework *F*_7_ depicted in Figure 7A. We describe how the four SCF2 extensions of *F*_7_ can be computed using the above algorithm. First, we delete the self-attacking argument *i*. Then, we divide the resulting AF into SCCs as depicted in Figure 7B. The only initial SCC is {*a, b, c, d, e, f*}, so in step 4 of the algorithm, we choose *C* to be this SCC. Now in step 5, we have two choices:

- We can choose *A* = {*b, d, f*}. In this case, we delete arguments *a*, *b*, *c*, *d*, *e*, *f*, and *j* from *F*_7_. We return to step 3, an divide the AF into SCCs, as depicted in Figure 7C. There are two initial SCCs, {*g*} and {*k*}. No matter which one we choose first, in the next step, we will have to choose *A* to be the completely chosen *SCC*. We then have one more iteration, in which we choose the set from {*g*} and {*k*} that we did not choose previously. Finally, the set *E* is {*b, d, f, g, k*}.
- We can choose *A* = {*a, c, e*}. In this case, we delete arguments *a*, *b*, *c*, *d*, *e*, *f*, and *g* from *F*_7_. We return to step 3, an divide the AF into SCCs, as depicted in Figure 7D. Now there are two initial SCCs, {*h*} and {*j, k, l*}. Again, it does not matter in which order we choose them. Suppose we first choose *h*. Then, *h* gets added to *E* and deleted. In the final iteration, we need to choose the SCC {*j, k, l*}. Here, we can choose *A* to be {*j*}, {*k*}, or {*l*}. This gives rise to three possible values for the constructed extension, {*a, c, e, h, j*}, {*a, c, e, h, k*}, and {*a, c, e, h, l*}.

In order to allow readers to develop an intuition for how the SCF2 semantics behaves and how it differs from other semantics, we present in Table 1 the extensions of all example AFs considered in Section 3 according to the SCF2 semantics and all semantics introduced in Section 2.

**Table 1:** Extensions of example AFs according to SCF2 and the semantics introduced in Section 2.

**Semantics**	***F*_1_**	***F*_2_**	***F*_3_**	***F*_4_**	***F*_5_**	***F*_6_**
SCF2	{*a*}, {*b*}, {*c*}	{*a, c, e*}, {*b, d, f*}	{*a*}	{*b*}	{*a*}	{*a, c*}
CF2	{*a*}, {*b*}, {*c*}	{*a, d*}, {*b, e*}, {*c, f*}, {*a, c, e*}, {*b, d, f*}	{*a*}, {*b*}	{*b*}	{*a*}	{*a, c*}, {*a, d*}, {*b, d*}
Naive	{*a*}, {*b*}, {*c*}	{*a, d*}, {*b, e*}, {*c, f*}, {*a, c, e*}, {*b, d, f*}	{*a*}, {*b*}	{*b*}, {*c*}	{*a*}, {*b*}	{*a, c*}, {*a, d*}, {*b, d*}
Stage2	{*a*}	{*a, c, e*}, {*b, d, f*}	{*a*}	{*b*}	{*a*}	{*a, c*}, {*a, d*}, {*b, d*}
Stage	{*a*}	{*a, c, e*}, {*b, d, f*}	{*a*}	{*b*}	{*a*}, {*b*}	{*a, c*}, {*a, d*}, {*b, d*}
Complete	∅, {*a*}	∅, {*a, c, e*}, {*b, d, f*}	∅, {*a*}	∅	{*a*}	∅
Stable	{*a*}	{*a, c, e*}, {*b, d, f*}	{*a*}	−	−	−
Grounded	∅	∅	∅	∅	{*a*}	∅
Preferred	{*a*}	{*a, c, e*}, {*b, d, f*}	{*a*}	∅	{*a*}	∅
Semi-stable	{*a*}	{*a, c, e*}, {*b, d, f*}	{*a*}	∅	{*a*}	∅

### 4.2. Principle-based motivation for SCF2

As we will show below, SCF2 satisfies directionality, INRA, and SCOOC, which we have argued to be desirable principles when evaluating a semantics designed to correspond well to what humans would consider a rational judgment on the acceptability of arguments. The somewhat complex definition of SCF2 raises the question whether a simpler definition could also be enough to satisfy these three principles.

To approach this question systematically, we would like to point out that the definition of SCF2 contains three features that distinguish it from naive semantics: It starts by deleting all self-attacking arguments (the function nsa), it proceeds by applying the SCC-recursive scheme (the function scc), and within each SCC, it applies SCOOC-naive rather than naive semantics. If we consider each of these three features a switch that we can switch on or off, we have eight definitions of semantics, namely, naive, nsa(naive), SCOOC-naive, nsa(SCOOC-naive), scc(naive), nsa(scc(naive)), scc(SCOOC-naive), and nsa(scc(SCOOC-naive)). One can easily see that naive = nsa(naive), so these eight definitions define only seven different semantics, whose properties we now study in order to show that only SCF2 satisfies all three principles directionality, INRA, and SCOOC.

Furthermore, we also consider naivety and SCC-recursiveness, as these principles are important for getting a better picture of the behavior of SCF2 and allow us to conclude that SCF2 also satisfies several other principles studied in the literature, as we will discuss at the end of this section.

Table 2 shows which of these seven semantics satisfies which of these five principles (we use the standard name CF2 for scc(naive) and use the short name SCF2 to refer to nsa(scc(SCOOC-naive))). Note that SCF2 satisfies all five principles, while no other of these seven semantics satisfies all five principles or even just the three principles directionality, INRA, and SCOOC.

**Table 2:** Properties of SCF2 and six semantics that are related to it with respect the five principles considered in this article.

	**Naivety**	**Directionality**	**SCC-recursiveness**	**INRA**	**SCOOC**
naive = nsa(naive)	✓	×	×	✓	×
SCOOC-naive	✓	×	×	×	✓
nsa(SCOOC-naive)	✓	×	×	×	✓
CF2	✓	✓	✓	×	×
nsa(CF2)	✓	✓	✓	✓	×
scc(SCOOC-naive)	✓	✓	✓	×	✓
SCF2	✓	✓	✓	✓	✓

Thus, the complexity of the definition of SCF2 is not arbitrary but is required in the sense that all three differences between the SCF2 semantics and the naive semantics (which has a much simpler definition) are needed to satisfy the considered principles. In other words, removing any non-empty subset of these three differences from the definition of the semantics would result in a semantics that does not satisfy all the considered principles.

We will now prove that every AF has an SCF2 extension and that the SCF2 semantics satisfies the five principles listed in Table 2. Concerning the other entries of Table 2, the results for CF2 and naive in the first three rows have been established in the literature (Baroni and Giacomin, 2007; van der Torre and Vesic, 2018), some of the results concerning INRA and SCOOC have been shown in Section 3, and the remaining results are proven in Appendix 1.

First we need a lemma, whose rather long and technical proof can be found in Appendix 1.

LEMMA 4. SCOOC-naive semantics is SCC-semi-rich.

THEOREM 5. Every AF has at least one SCF2 extension.

PROOF. Lemma 4 implies that every single-SCC AF has a SCOOC-naive extension. This, together with the definition of the SCC recursive scheme, implies that every AF has at least 1 s (SCOOC-naive)-extension, and hence at least one SCF2 extension.     □

The proof of the following two theorems are in the appendix.

THEOREM 6. SCF2 satisfies naivety.

THEOREM 7. SCF2 satisfies directionality.

THEOREM 8. SCF2 satisfies SCC-recursiveness.

PROOF. From the definition of SCF2 it is immediately that SCF2 = scc(SCF2) and that, therefore, SCF2 is SCC-recursive with base function *BF*_*S*_(*F, C*): = SCF2(*F*).

     □

THEOREM 9. SCF2 satisfies SCOOC.

PROOF. Consider an AF *F*, an SCF2 extension *E* of *F*, and an argument *a* ∈ *Ar* such that no argument in {*a*}∪*a*^−^ is in an odd cycle and *E* ∩ *a*^−^ = ∅. Then by definition of SCF2 semantics, the moment the SCOOC-naive function is applied to a sub-framework of *F* containing *a*, we have *a* ∈ *E*. Consequently, *E* is strongly complete outside odd cycles.     □

THEOREM 10. SCF2 satisfies INRA.

PROOF. By Lemma 4, SCOOC-naive semantics is SCC-semi-rich. So, by Lemma 3 and the definition of SCF2 it follows that SCF2 satisfies INRA.     □

Concerning the other principles studied in the literature, SCF2 has almost the same properties as CF2, the only exception being the succinctness principle (van der Torre and Vesic, 2018). This is proven in Appendix 3. Most of the positive results follow directly from the results established above using logical relationships between principles that have been established in the literature (van der Torre and Vesic, 2018) – here, naivety and SCC-recursiveness play a crucial role.

## 5. Empirical cognitive studies

Rahwan et al. (2010) argued that artificial intelligence research will benefit from the interplay between logic and cognition and that; therefore, “logicians and computer scientists ought to give serious attention to cognitive plausibility when assessing formal models of reasoning, argumentation, and decision making.” Based on the observation that in the previous literature on formal argumentation theory, an example-based approach and a principle-based approach were used to motivate and validate argumentation semantics, they propose to complement these approaches by an *experiment-based approach* that takes into account empirical cognitive studies on how humans interpret and evaluate arguments. They made a first contribution to this new approach by presenting and discussing the results of two such studies that they conducted in order to test the cognitive plausibility of simple and floating reinstatement (Rahwan et al., 2010).

While the argumentation frameworks used in Rahwan et al.'s studies could not distinguish between preferred semantics and naive-based semantics like CF2, two more recent studies by Cramer and Guillaume (2018b, 2019) addressed this issue. Both of these studies made use of a group discussion methodology that is known to stimulate more rational thinking. According to the results of the first study (Cramer and Guillaume, 2018b), CF2, SCF2, stage, and stage2 semantics are significantly better predictors for human judgments on the acceptability of arguments than admissibility-based semantics like grounded, preferred, complete or semi-stable (all *p*-values < 0.001), but this study did not involve argumentation frameworks that allow distinguishing between CF2, SCF2, stage, and stage2 semantics.

According to the results of the second study (Cramer and Guillaume, 2019), SCF2, CF2, and grounded semantics are better predictors for human judgments on the acceptability of arguments than stage, stage2, preferred or semi-stable semantics (all *p* < 0.001). In addition, the results suggest that SCF2 is a better predictor than CF2 and grounded semantics, but the results are not significant.^2^ We will now explain these results in more depth.

As explained in Section 3, Dvořák and Gaggl (2016) critique a feature of CF2 semantics, namely, that in the case of a six-cycle, as depicted in Figure 2, CF2 allows two opposite arguments (e.g., *a* and *d*) to be accepted together. The second study by Cramer and Guillaume (2019) confirms that this criticism is in line with human judgments of argument acceptability. We briefly summarize the data on which this judgment is made (a more detailed explanation can by found in Cramer and Guillaume, 2019): Based on the overall responses of the participants in the study, Cramer and Guillaume pointed out that 12 of the 61 participants of their study have a high frequency of incoherent responses, so that they disconsider them from the further analysis. Among the remaining 49 participants, 22 follow a simple cognitive strategy of marking arguments as *Undecided* whenever there is a reason for doubt (in line with the grounded semantics), while 27 participants do not follow this strategy. Cramer and Guillaume called these 27 participants the *coherent non-grounded participants*.

In the case of 11 out of the 12 argumentation frameworks considered in the study, the majority of these 27 coherent non-grounded participants make judgments that are in line with CF2 semantics. The only exception to this is an argumentation framework involving a six-cycle, in which only 33% of the coherent non-grounded participants make a judgment in line with CF2 semantics, while 60% make a judgments that are similar in line with SCF2, stage2, preferred and semi-stable semantics.

Dvořák and Gaggl (2016) themselves had used this criticism against CF2 to motivate their stage2 semantics, but in the study by Cramer and Guillaume (2019), stage2 performed worse than CF2, as all other AFs in which stage2 and CF2 had different predictions were evaluated by most participants (including most coherent non-grounded participants) more in line with CF2 than with stage2.

In combination with the principle-based argument for SCF2 presented in the previous two sections, this provides additional support for our claim that SCF2 corresponds well to what humans consider a rational judgment on the acceptability of arguments.

## 6. Related work

The principle-based analysis of argumentation semantics was initiated by Baroni and Giacomin (2007) to choose among the many extension-based argumentation semantics that have been proposed in the formal argumentation literature. The handbook chapter of van der Torre and Vesic (2018) gives a classification of 15 alternatives for argumentation semantics using 27 principles discussed in the literature on abstract argumentation. Dvořák and Gaggl (2016) introduced stage2 semantics by showing how it satisfies various desirable properties, similar to how we motivate SCF2 semantics in this article.

Moreover, additional extension-based argumentation semantics and principles have been proposed by various authors. For example, Besnard et al. (2016) introduced a system for specifying semantics in abstract argumentation called SESAME. Moreover, many principles have been proposed for alternative semantics of argumentation frameworks, such as ranking semantics (Amgoud and Ben-Naim, 2013), and for extended argumentation frameworks, for example, for abstract dialectical frameworks (Brewka et al., 2018).

The principle of Irrelevance of Necessarily Rejected Arguments is closely related to the well-studied area of dynamics of argumentation, in which also various principles have been proposed which are closely related to INRA. Cayrol et al. (2008) were maybe the first to study revision of frameworks using a principle-based analysis, and they have been related to notions of equivalence (Baumann, 2012; Oikarinen and Woltran, 2011). (Boella et al., 2009) defined principles for abstracting (i.e., removing) an argument, and (Rienstra et al., 2015) defined a variety of persistence and monotony properties for argumentation semantics. Our INRA principle is inspired by and closely related to the *skeptical IO monotony principle* they define. The difference is that their principle considers adding an attack rather than removing an argument.

After the INRA principle was proposed in the workshop article (Cramer and van der Torre, 2019) on which the current article is based, Cramer and Spörl (2021) studied the INRA principle in connection with the notion of admissibility and developed a new admissibility-based semantics—the *choice-preferred semantics*—that satisfies INRA.

The study of semantics and principles for abstract argumentation remains an active area of research. During the past few years, various new semantics have been proposed that are neither admissibility based nor naive based (Dvorák et al., 2022). These semantics were mainly motivated by the idea that self-attacking arguments should not affect the acceptance of other arguments, which has been called ambiguity blocking or undecidedness blocking. For these and other semantics, it remains to be checked whether they satisfy the INRA and SCOOC principles introduced in this article.

In addition to the cognitive studies on formal argumentation that are already mentioned in Section 5, several other such studies have been conducted. Cerutti et al. (2021) give an overview of empirical cognitive studies about formal argumentation. Concerning investigations into the relation between argumentation semantics from abstract argumentation on the one hand and human argument evaluation on the other, this overview article only lists the articles already mentioned in Section 5. The remaining articles mentioned in the overview article by Cerutti et al. are concerned with argumentation formalisms from other areas of formal argumentation like structured argumentation [e.g., Cerutti et al. (2014) and Yu et al. (2018)] as well as probabilistic and bipolar argumentation (e.g., Polberg and Hunter, 2018). Since these studies are about other areas of formal argumentation, they are not directly relevant to the research question addressed in this article. Concerning studies on abstract argumentation, there is also a recent article by Guillaume et al. (2022) that gives a more detailed analysis of the results from the study first presented in Cramer and Guillaume (2018b).

## 7. Conclusion and future work

Motivated by empirical cognitive studies on argumentation semantics, we have introduced a new naive-based argumentation semantics called SCF2. A principle-based analysis shows that it has two distinguishing features:

1. If an argument is attacked by all extensions, then it can never be used in a dialogue and, therefore, it has no effect on the acceptance of other arguments. We call it *Irrelevance of Necessarily Rejected Arguments*.
2. Within each extension, if none of the attackers of an argument is accepted and the argument is not involved in a paradoxical relation, then the argument is accepted. We define paradoxicality as being part of an odd cycle, and we call this principle *Strong Completeness Outside Odd Cycles*.

We have argued that these features, together with further satisfied principles and the findings from empirical cognitive studies, make SCF2 a good candidate for an argumentation semantics that corresponds well to what humans consider a rational judgment on the acceptability of arguments.

Though many results have been obtained—some of them listed in the appendix—there is also some work left to be done. First of all, for a few principles discussed in the literature, it still needs to be shown whether they hold for SCF2 or not. Moreover, dialogue-based decision procedures must be defined, and the complexity of the various decision problems must be established. Finally, an extension toward structured argumentation should be investigated.

## Data availability statement

The original contributions presented in the study are included in the article/Supplementary material, further inquiries can be directed to the corresponding author.

## Author contributions

While MC has done most of the work, LT helped with proving a significant amount of the formal results listed in Appendix 3 and with improving the wording and presentation of the whole paper. Both authors contributed to the article and approved the submitted version.

## Footnotes

^1^Note that the deletion of arguments mentioned in this principle only concerns the procedure for deciding which arguments are accepted according to the SCF2 argumentation semantics. In applications of the SCF2 semantics to structured argumentation or to the formal study of dialogues, the deletion of arguments would not happen at the level of argument construction but only at the level of argument evaluation. So, even arguments that are rejected by everyone could influence the dynamics of argument construction by participants of a dialogue.

^2^While the SCF2 semantics had not yet been proposed at the time when this study and the two studies mentioned before were conducted, the design of the studies was such that they were not specifically tailored toward the semantics that the results were compared to in the articles about the studies. In other words, the results of the studies can be equally compared to any argumentation semantics whatsoever. Here, we compare them to the SCF2 semantics in addition to the semantics already considered in the original articles about the studies.
