# Supplementary material for: An argumentation semantics for rational human evaluation of arguments
Source: Front Artif Intell. 2023 Mar 23;6:1045663. doi: 10.3389/frai.2023.1045663 (PMC10076807; doi:10.3389/frai.2023.1045663)
Supplement: Supplementary file 1 [file Data_Sheet_1.pdf]

APPENDICES

1 PROOFS OF LEMMAS AND THEOREMS FROM THE PAPER

PROOF OF LEMMA 3. We first prove part (a) of this lemma and then show how the proof can be adapted to prove part (b).

Let σ be an SCC-rich semantics. Let $F = \langle Ar, att \rangle$ be an AF and let $a \in Ar$ be an argument such that for every $E \in \mathrm{scc}(\sigma)(F)$, E attacks a. We need to show that $\mathrm{scc}(\sigma)(F) = \mathrm{scc}(\sigma)(F^{-a})$.

Note that F must have more than one SCC, because otherwise the SCC-richness of σ would imply that there is an extension that does not attack a. We now distinguish two cases:

Case (i): $|SCCs(F^{-a})| = 1$. In this case, F has two SCCs, namely $\{a\}$ and $Ar \setminus \{a\}$. Since a is attacked by every extension, it must be attacked from some argument in $Ar \setminus \{a\}$, so a does not attack any argument in $Ar \setminus \{a\}$. So $Ar \setminus \{a\}$ is the initial SCC of F and every extension of F^{-a} contains an argument attacking a. So $\mathrm{scc}(\sigma)(F^{-a}) = \sigma(F^{-a}) = \mathrm{scc}(\sigma)(F)$.

Case (ii): $|SCCs(F^{-a})| > 1$. In this case, we prove the result by an induction over the number of arguments in F, so we may assume as induction hypothesis that it holds for strict subframeworks of F. Let C_a denote the SCC of F that contains a.

First we show that $\mathrm{scc}(\sigma)(F) \subseteq \mathrm{scc}(\sigma)(F^{-a})$. Let $S \in \mathrm{scc}(\sigma)(F)$. Let $C \in SCCs(F)$. Since $|SCCs(F)| > 1$, it follows by Definition 5 that $S \cap C$ is an $\mathrm{scc}(\sigma)$-extension of $F|_{C \setminus D_F(S)}$. By the induction hypothesis, $S \cap C$ is an $\mathrm{scc}(\sigma)$-extension of $F|_{C \setminus (D_F(S) \cup \{a\})}$. So we have established that for each $C \in SCCs(F)$, $S \cap C$ is an $\mathrm{scc}(\sigma)$-extension of $F|_{C \setminus (D_F(S) \cup \{a\})}$ (1).

We need to show that $S \in \mathrm{scc}(\sigma)(F^{-a})$. Let $C' \in SCCs(F^{-a})$. By Definition 5 and the fact that $|SCCs(F^{-a})| > 1$, it is enough to show that this arbitrarily chosen $C' \in SCCs(F^{-a})$ satisfies the following property:

(*) $S \cap C'$ is an $\mathrm{scc}(\sigma)$-extension of $F^{-a}|_{C'} \setminus D_{F^{-a}}(S)$.

Note that either $C' \in SCCs(F)$ or that $C' \subseteq C_a$. We consider these two cases separately.

If $C' \in SCCs(F)$, then $F^{-a}|_C \setminus D_{F^{-a}}(S) = F|_{C \setminus (D_F(S) \cup \{a\})}$, so the required property (*) directly follows from (1).

If $C' \subseteq C_a$, we show (*) by making the case distinction from the definition of $\mathrm{scc}(\sigma)$:

Case 1: $|SCCs(F|_{C_a} \setminus (D_F(S) \cup \{a\}))| = 1$. Then $|SCCs(F^{-a}|_{C_a})| = 1$, so the single SCC of $F|_{C_a} \setminus (D_F(S) \cup \{a\})$ must either be fully contained in C' or disjoint from C'. In the first case, $C' = C_a \setminus \{a\}$, so $S \cap C' = S \cap C_a$ $F|_{C_a} \setminus (D_F(S) \cup \{a\}) = F^{-a}|_{C'} \setminus D_{F^{-a}}(S)$. Therefore (1) applied to C_a implies that property (*) holds. In the second case, $C' \subseteq D_{F^{-a}}(S)$ and $S \cap C' = \emptyset$, so (*) holds because the empty set is a $\mathrm{scc}(\sigma)$-extension of the empty framework.

Case 2: $|SCCs(F|_{C_a} \setminus (D_F(S) \cup \{a\}))| > 1$. Then by Definition 5, for each $C^* \in SCCs(F|_{C_a} \setminus (D_F(S) \cup \{a\}))$, $S \cap C^*$ is an $\mathrm{scc}(\sigma)$-extension of $F|_{C^*} \setminus D_{F|_{C_a} \setminus (D_F(S) \cup \{a\})}(S)$. Let $C'' \in SCCs(F|_{C'} \setminus D_F(S))$ (2). Then $C'' \in SCCs(F|_{C_a} \setminus (D_F(S) \cup \{a\}))$, because $F|_{C'} \setminus D_F(S) \subseteq F|_{C_a} \setminus (D_F(S) \cup \{a\})$ (note that C'' cannot be expanded to a larger SCC in $F|_{C_a} \setminus (D_F(S) \cup \{a\})$, because C' is an SCC of F and would therefore have to contain this expansion of C''). Now (2) together with Definition 5 implies property (*).

This concludes the proof that $S \in \text{scc}(\sigma)(F^{-a})$ and thus that $\text{scc}(\sigma)(F) \subseteq \text{scc}(\sigma)(F^{-a})$ The proof that $\text{scc}(\sigma)(F^{-a}) \subseteq \text{scc}(\sigma)(F)$ works similarly.

The main part of the proof of part (b) works similarly, but the beginning is a bit different:

Let σ be an SCC-semi-rich semantics. Let $F = \langle Ar, att\rangle$ be an AF and let $a \in Ar$ be an argument such that for every $E \in \text{nsa}(\text{scc}(\sigma))(F)$, $a \notin E$. We need to show that $\text{nsa}(\text{scc}(\sigma))(F) = \text{nsa}(\text{scc}(\sigma))(F^{-a})$. Define $F' := NSA(F)$. We need to show that $\text{scc}(\sigma)(F) = \text{scc}(\sigma)(F^{-a})$. If a is not an argument in F', then the result trivially holds. So suppose a is in F'. Note that F' must have more than one SCC, because otherwise the SCC-semi-richness of σ would imply that there is an extension that does not attack a.

Now we continue as in the proof of part (a), just with F' in place of F. □

PROOF OF LEMMA 4. Let $F = \langle Ar, att\rangle$ be an AF such that $|SCCs(F) = 1|$ and let $c \in Ar$ be an argument such that $(c, c) \notin att$. We prove the lemma by induction over $|Ar|$, so we assume for the inductive hypothesis that it holds for strict subframeworks of F. We need to find an SCF2-extension E of F such that E does not attack c. We do this by specifying a non-deterministic procedure to construct such an extension. The constructed extension will be constructed in such a way that it must contain c, which implies that it does not attack c.

At each step k of the procedure, we identify a set E_k of arguments to be included in E and a set $\bar{E}_k$ of arguments for which we rule out that they may be in E. We define U_k to be set of arguments a such that it is not yet determined by step k whether a is included in E or not; formally, $U_k := Ar \setminus \bigcup_{i<k}(E_k \cup \bar{E}_k)$. We set $\bar{E}_0 := \{a \in Ar \mid (a, a) \in att\}$ and $E_0 := \{c\}$. Let $k > 0$. We set $\bar{E}_k := \{a \in Ar \mid \text{some argument in } E_{k-1} \text{ attacks } a \text{ or is attacked by } a\}$. For the definition of E_k we have a case-distinction:

1. If $E_{k-1} \neq \emptyset$, we define $E_k := E_k^1 \cup E_k^2$, where $E_k^1 := \{a \in U_k \mid \text{all attakers of } a \text{ are in some } \bar{E}_i \text{ for } i < k\}$, and $E_k^2 := \{a \in U_k \mid \text{for some } b \in \bar{E}_{k-1} \text{ that is not in an odd cycle and whose attackers are not in an odd cycle and not in any } E_i \text{ for } i < k, a \text{ attacks } b\}$.
2. If $E_{k-1} = \emptyset$ and $U_k \neq \emptyset$, we set E_k to be a SCOOC-naive extension of an unattacked SCC of U_k (which exists and is non-empty by inductive hypothesis and by the fact that U_k does not contain self-attacking arguments, as these are all in $\bar{E}_0$).
3. If $E_{k-1} = \emptyset$ and $U_k = \emptyset$, the procedure stops.

When the procedure stops at step k, we set E to be the union of all E_i for $i < k$. Note that once case 2 is applied, only case 2 can be applied. Let n denote the step at which case 2 is first applied. Note that a simple proof by induction establishes that whenever $a \in E_k^2$ for $k < n$, then there is an even-length path from a to c (1). This can be generalized to the statement that whenever $a \in E_k$ for $k < n$, there are a', k' such that $a' \in \bar{E}_{k'}$ and there is an odd-length att-path from a' to c and from a' to a (2).

$a \in E$ because $a \in E_0$. It is easy to see from the construction of E that E is strongly complete outside odd cycles and that adding an argument to E creates a conflict within E. So all we still need to prove is that E is conflict-free. Suppose for a contradiction that $(a_1, a_2) \in att$ for $a_1, a_2 \in E$. From the definition of $\bar{E}_k$ for $k > 0$ it follows that there is an i such that $a_1, a_2 \in E_i$. Clearly this is not the case if E_i was defined according to case 2 of the definition of E_k, so $k < n$. By the definition of E_k^1, it furthermore follows that $a_2 \notin E_i^1$. So $a_2 \in E_i^2$, i.e. by property (1) there is an even-length att-path p_0 from a_2 to c. Furthermore, by property (2) there is an a_1' such that there is an odd-length att-path p_1 from a_1' to c and from a_1' to a_1. Since

$|\mathit{SCCs}(F) = 1|$, there is a path p_2 from a_2 to a_1'. The length of path p_2 must be even, because if it were odd, then concatenating p_2 with the odd-length path p_1 and the attack from a_1 to a_2 would result in an odd cycle through a_2, which cannot exist as $a_2 \in E_i^2$. Since $|\mathit{SCCs}(F) = 1|$, there is a path p_3 from c to a_2. If the length of p_3 is odd, then p_3 concatenated with the even-length path p_0 is an odd cycle through a_2. If the length of p_3 is even, the p_3 concatenated with the even-length path p_2 and the attack from a_1' to c is an odd cycle through a_2. so in either case, there is an odd cycle through a_2, which contradicts the fact that $a_2 \in E_i^2$. This completes our proof by contradiction that E is conflict-free. □

PROOF OF THEOREM 6. From the definition of scc and SCOOC-naive, one can easily see that every scc(SCOOC-naive)-extension and hence every SCF2 extension is conflict-free.

Now suppose for a contradiction that E is an SCF2-extension of an AF F and that S is a conflict-free strict superset of E, i.e. there exists an $a \in S \setminus E$. By the definition of SCF2, a must have been excluded from E for one of three reasons, either because a attacks itself and was thus filtered out by the nsa function, or because a was filtered out in the SCC-recursive scheme for being attacked by an element of E from another SCC, or because a was not in the SCOOC-naive-extension E' chosen when considering a single SCC of a subframework F' of F. The first two cases can immediately be ruled out as they would imply that S is not conflict-free. In the third case, $E' \cup \{a\}$ would be conflict-free and would just like E' be strongly-complete outside odd cycles, contradicting the maximality condition in the definition of SCOOC-naive. □

PROOF OF THEOREM 7. Consider an AF F and an unattacked set U. Define $F' = (\mathit{Ar}', \mathit{att}')$ to be $\mathit{NSA}(F)$. Note that U is unattacked in F' and that therefore $\mathit{SCCs}(F') = \mathit{SCCs}(F'|_U) \cup \mathit{SCCs}(F'|_{\mathit{Ar}'\setminus U})$ (1).

First, we show that if $E_1 \in$ SCF2$(F|_U)$ then there exists $E_2 \in$ SCF2(F) such that $E_1 = E_2 \cap U$. Assume $E_1 \in$ SCF2$(F|_U)$. Then $E_1 \in$ scc(SCOOC-naive)$(F'|_U)$, i.e. for each $C \in \mathit{SCCs}(F'|_U)$, $E_1 \cap C$ is an scc(SCOOC-naive)-extension of $F'|_C \setminus D_{F'}(E_1)$ (2). By Theorem 5, $F'_{\mathit{Ar}\setminus U} \setminus D_{F'}(E_1)$ has an SCF2 extension, say E. Define $E_2 := E_1 \cup E$. Note that $E_1 = E_2 \cap U$, so it is enough to show that E_2 is an SCF2-extension of F. Since $F' = \mathit{NSA}(F')$, E is an scc(SCOOC-naive)-extension of $F'_{\mathit{Ar}\setminus U} \setminus D_{F'}(E_1)$, i.e. for each $C \in \mathit{SCCs}(F'|_{\mathit{Ar}\setminus U})$, $E \cap C$ is an scc(SCOOC-naive)-extension of $F'|_C \setminus D_{F'}(E)$. This together with (1) and (2) implies that E_2 is an scc(SCOOC-naive)-extension of F', i.e. that E_2 is an SCF2-extension of F, as required.

Now, we show that if $E \in$ SCF2(F) then $E \cap U \in$ SCF2$(F|_U)$. Assume $E \in$ SCF2(F). Then for each $C \in \mathit{SCCs}(F')$, $E \cap C$ is an scc(SCOOC-naive)-extension of $F'|_C \setminus D_F(A)$. By (1), it follows that $E \cap U$ is an scc(SCOOC-naive)-extension of F', i.e. that $E \cap U$ is an SCF2-extension of F, as required. □

2 DEFINITIONS OF FURTHER PRINCIPLES

In this appendix we define further principles studied by van der Torre and Vesic (2018), as we will discuss the properties of SCF2 with respect to these principles in Appendix 3.

DEFINITION 24 (Isomorphic argumentation frameworks). *Two argumentation frameworks* $F_1 = (\mathit{Ar}_1, \mathit{att}_1)$ *and* $F_2 = (\mathit{Ar}_2, \mathit{att}_2)$ *are* isomorphic *if and only if there exists a bijective function* $m : \mathit{Ar}_1 \to \mathit{Ar}_2$*, such that* $(a, b) \in \mathit{att}_1$ *if and only if* $(m(a), m(b)) \in \mathit{att}_2$*. This is denoted by* $F_1 \doteq_m F_2$*.*

DEFINITION 25 (Language independence). *A semantics* σ *satisfies the* language independence *principle if and only if for every two argumentation frameworks* F_1 *and* F_2*, if* $F_1 \doteq_m F_2$ *then* $\sigma(F_2) = \{m(E) \mid E \in \sigma(F_1)\}$*.*

DEFINITION 26 (Conflict-freeness). *A semantics σ satisfies the* conflict-freeness *principle if and only if for every argumentation framework F, for every $E \in \sigma(F)$, E is conflict-free set in F.*

DEFINITION 27 (Defence). *A semantics σ satisfies the* defence *principle if and only if for every argumentation framework F, for every $E \in \sigma(F)$, for every $a \in E$, E defends a.*

DEFINITION 28 (Admissibility). *A semantics σ satisfies the* admissibility *principle if and only if for every argumentation framework F, every $E \in \sigma(F)$ is admissible in F.*

DEFINITION 29 (Strong admissibility). *A semantics σ satisfies the* strong admissibility *principle if and only if for every argumentation framework F, for every $E \in \sigma(F)$ it holds that $a \in E$ implies that E strongly defends a.*

DEFINITION 30 (Indirect conflict-freeness). *A semantics σ satisfies the* indirect conflict-freeness *principle if and only if for every argumentation framework F, for every $E \in \sigma(F)$, E is without indirect conflicts in F.*

DEFINITION 31 (Reinstatement). *A semantics σ satisfies the* reinstatement *principle if and only if for every argumentation framework F, for every $E \in \sigma(F)$, for every $a \in Ar$ it holds that if E defends a then $a \in E$.*

DEFINITION 32 (Weak reinstatement). *A semantics σ satisfies the* weak reinstatement *principle if and only if for every argumentation framework F, for every $E \in \sigma(F)$ it holds that*

$$E \textit{ strongly defends } a \textit{ implies } a \in E.$$

DEFINITION 33 (CF-reinstatement). *A semantics σ satisfies the* CF-reinstatement *principle if and only if for every argumentation framework F, for every $E \in \sigma(F)$, for every $a \in Ar$ it holds that if E defends a and $E \cup \{a\}$ is conflict-free then $a \in E$.*

DEFINITION 34 (I-maximality). *A semantics σ satisfies the* I-maximality *principle if and only if for every argumentation framework F, for every $E_1, E_2 \in \sigma(F)$, if $E_1 \subseteq E_2$ then $E_1 = E_2$.*

DEFINITION 35 (Allowing abstention). *A semantics σ satisfies the* allowing abstention *principle if and only if for every argumentation framework F, for every $a \in Ar$, if there exist two extensions $E_1, E_2 \in \sigma(F)$ such that $a \in E_1$ and $a \in E_2^+$ then there exists an extension $E_3 \in \sigma(F)$ such that $a \notin (E_3 \cup E_3^+)$.*

DEFINITION 36 (Disjoint argumentation frameworks). *Two argumentation frameworks $F_1 = (Ar_1, att_1)$ and $F_2 = (Ar_2, att_2)$ are disjoint if and only if $Ar_1 \cap Ar_2 = \emptyset$.*

DEFINITION 37 (Contaminating). *An argumentation framework $F_\star$ is contaminating for a semantics σ if and only if for every argumentation framework F disjoint from $F_\star$ it holds that $\sigma(F_\star \cup F) = \sigma(F_\star)$.*

DEFINITION 38 (Crash resistance). *A semantics σ satisfies the* crash resistance *principle if and only if there are no contaminating argumentation frameworks for σ.*

DEFINITION 39 (Isolated set of arguments). *Let $F = (Ar, att)$ be an argumentation framework. A set $S \subseteq Ar$ is isolated in F if and only if*

$$((S \times (Ar \setminus S)) \cup ((Ar \setminus S) \times S)) \cap att = \emptyset.$$

DEFINITION 40 (Non-interference). *A semantics σ satisfies the* non-interference *principle if and only if for every argumentation framework F, for every set of arguments S isolated in F it holds that $\sigma(F|_S) = \{E \cap S \mid E \in \sigma(F)\}$.*

DEFINITION 41 (Unattacked arguments). *Given an argumentation framework $F = (Ar, att)$, a set U is* unattacked *if and only if there exists no $a \in Ar \setminus U$ such that a attacks U. The set of unattacked sets in F is denoted $US(F)$.*

DEFINITION 42 (Weak directionality). *A semantics σ satisfies the* weak directionality *principle if and only if for every argumentation framework F, for every $U \in US(F)$, it holds that $\sigma(F|_U) \supseteq \{E \cap U \mid E \in \sigma(F)\}$.*

DEFINITION 43 (Semi-directionality). *A semantics σ satisfies the* semi-directionality *principle if and only if for every argumentation framework F, for every $U \in US(F)$, it holds that $\sigma(F|_U) \subseteq \{E \cap U \mid E \in \sigma(F)\}$.*

DEFINITION 44 ($\preceq^E_W$). *Let Ext_1 and Ext_2 be two sets of sets of arguments. We say that $Ext_1 \preceq^E_W Ext_2$ if and only if*

$$\text{for every } E_2 \in Ext_2, \text{ there exists } E_1 \in Ext_1 \text{ such that } E_1 \subseteq E_2.$$

DEFINITION 45 ($\preceq^E_S$). *Let Ext_1 and Ext_2 be two sets of sets of arguments. We say that $Ext_1 \preceq^E_S Ext_2$ if and only if $Ext_1 \preceq^E_W Ext_2$ and*

$$\text{for every } E_1 \in Ext_1, \text{ there exists } E_2 \in Ext_2 \text{ such that } E_1 \subseteq E_2.$$

DEFINITION 46 ($\preceq^A$). *Given an argumentation framework $F = (Ar, att)$, the conflict set is defined as $CONF(F) = \{(a,b) \in Ar \times Ar \mid (a,b) \in att \text{ or } (b,a) \in att\}$. Given two argumentation frameworks $F_1 = (Ar_1, att_1)$ and $F_2 = (Ar_2, att_2)$, we say that $F_1 \preceq^A F_2$ if and only if $CONF(F_1) = CONF(F_2)$ and $att_2 \subseteq att_1$.*

DEFINITION 47 (Skepticism adequacy). *Given a skepticism relation $\prec^E$ between sets of sets of arguments, a semantics σ satisfies the $\preceq^E$-*skepticism adequacy *principle if and only if for every two argumentation frameworks F and F' such that $F \preceq^A F'$ it holds that $\sigma(F) \preceq^E \sigma(F')$.*

DEFINITION 48 (Strong equivalence). *Two argumentation frameworks F_1 and F_2 are strongly equivalent with respect to semantics σ, in symbols $F_1 \equiv^\sigma_s F_2$ if and only if for each argumentation framework F_3, $\sigma(F_1 \cup F_3) = \sigma(F_2 \cup F_3)$.*

DEFINITION 49 (Redundant attack). *Let $F = (Ar, att)$ be an argumentation framework and σ and semantics. Attack $(a,b) \in att$ is said to be redundant in F with respect to σ if and only if for all argumentation frameworks F' such that $F \subseteq F'$ we have $\sigma(F') = \sigma(F' \setminus (a,b))$.*

DEFINITION 50 (Succinctness). *A semantics σ satisfies the* succinctness *principle if and only if no argumentation framework contains a redundant attack with respect to σ.*

DEFINITION 51 ($Pairs$). *Given a set of extensions $\mathcal{S} = \{E_1, \ldots, E_n\}$, we define*

$$Pairs(\mathcal{S}) = \{(a,b) \mid \text{ there exists } E_i \in \mathcal{S} \text{ such that } \{a,b\} \subseteq E_i\}.$$

DEFINITION 52 (Tightness). *A set of extensions $\mathcal{S} = \{E_1, \ldots, E_n\}$ is tight if and only if for every extension E_i and for every $a \in Ar$ that appears in at least one extension from $\mathcal{S}$ it holds that if $E_i \cup \{a\} \notin \mathcal{S}$ then there exists $b \in E_i$ such that $(a,b) \notin Pairs(\mathcal{S})$.*

A semantics σ satisfies the tightness *principle if and only if for every argumentation framework F, $\sigma(F)$ is tight.*

DEFINITION 53 (Conflict-sensitiveness). *A set of extensions $\mathcal{S} = \{E_1, \ldots, E_n\}$ is conflict-sensitive if and only if for every two extensions E_i, E_j such that $E_i \cup E_j \notin \mathcal{S}$ it holds that there exist $a, b \in E_i \cup E_j$ such that $(a, b) \notin Pairs_{\mathcal{S}}$.*

A semantics σ satisfies the conflict-sensitiveness *principle if and only if for every argumentation framework F, $\sigma(F)$ is conflict-sensitive.*

DEFINITION 54 (Completion set). *Given a set of extensions $\mathcal{S} = \{E_1, \ldots, E_n\}$ and a set of arguments E, set E' is a completion set of E in $\mathcal{S}$ if and only if E' is a minimal for $\subseteq$ set such that $E' \in \mathcal{S}$ and $E \subseteq E'$.*

DEFINITION 55 (Com-closure). *A set of extensions $\mathcal{S} = \{E_1, \ldots, E_n\}$ is com-closed if and only if for every $T \subseteq \mathcal{S}$ the following holds: if $(a, b) \in Pairs_{\mathcal{S}}$ for each $a, b \in \cup_{E_i \in T} E_i$, then $\cup_{E_i \in T} E_i$ has a unique completion set in $\mathcal{S}$.*

A semantics σ satisfies the com-closure *principle if and only if for every argumentation framework F, $\sigma(F)$ is com-closed.*

3 ADDITIONAL RESULTS

We first establish those results shown in Table 2 that were neither shown in the paper nor in Section 1 of this supplementary material. We start with the positive results.

THEOREM 11. *SCOOC-naive, nsa(SCOOC-naive), CF2, nsa(CF2) and scc(SCOOC-naive) satisfy Naivety.*

PROOF. Suppose E is a SCOOC-naive extension of F, and suppose for a contradiction that $a \notin E$ and $E \cup \{a\}$ is conflict-free. Since E is strongly complete outside odd cycles, so is $E \cup \{a\}$, contradicting the fact that E is a maximal conflict free set that is strongly complete outside odd cycles. Thus the SCOOC-naive semantics satisfies Naivety.

This immediately implies that also nsa(SCOOC-naive) satisfies Naivety.

CF2 was proven to satisfy Naivety by van der Torre and Vesic (2018). This immediately implies that also nsa(CF2) satisfies Naivety.

The proof that scc(SCOOC-naive) satisfies Naivety is the same as the second paragraph of the proof that SCF2 satisfies Naivety (Theorem 6), just replacing the name “SCF2” by “scc(SCOOC-naive)”. □

THEOREM 12. *CF2, nsa(CF2) and scc(SCOOC-naive) satisfy Directionality.*

PROOF. The result for CF2 has been shown by Baroni and Giacomin (2007) and directly implies the result for nsa(CF2). The proof for scc(SCOOC-naive) works just like the proof that SCF2 satisfies Directionality (Theorem 7). □

THEOREM 13. *CF2, nsa(CF2) and scc(SCOOC-naive) satisfy SCC-recursiveness.*

PROOF. Immediate from definitions. □

THEOREM 14. *SCOOC-naive, nsa(SCOOC-naive) and scc(SCOOC-naive) satisfy SCOOC.*

PROOF. For SCOOC-naive and nsa(SCOOC-naive), this follows directly from the definitions. As for scc(SCOOC-naive), the proof that SCF2 satisfies SCOOC (Theorem 9) also establishes that scc(SCOOC-naive) satisfies SCOOC. □

We now prove the negative results shown in Table 2.

THEOREM 15. *Naive semantics violates Directionality, SCC-recursiveness and SCOOC.*

PROOF. A counterexample to all three principles is shown in Figure 1.

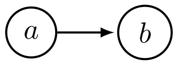

Figure 1. Naive semantics violates Directionality, because a is not in the extension $\{b\}$, even though it is in the only extension of the unattacked subframework $\{a\}$. Naive semantics violates SCC-recursiveness, because $\{b\}$ is an extension even though the acceptence of a in the SCC $\{a\}$ should force b to be out. Naive semantics violates SCOOC, because a is not in the extension $\{b\}$, even though it is not in an odd cycle, it does not have an attacker in an odd cycle and does not have an attacker in the extension $\{b\}$.

THEOREM 16. *SCOOC-naive semantics violates Directionality, SCC-recursiveness and INRA.*

PROOF. A counterexample to all three principles is shown in Figure 2.

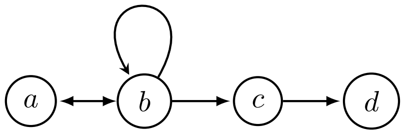

Figure 2. SCOOC-naive semantics violates Directionality, because c is not in the extension $\{a, d\}$, even though it is in the only extension of the unattacked subframework $\{a, b, c\}$. SCOOC-naive semantics violates SCC-recursiveness, because $\{c\}$ is not in the extension $\{a, d\}$ even though the rejection of b in the SCC $\{a, b\}$ should force c to be in. SCOOC-naive semantics violates INRA, because b is attacked by both extensions ($\{a, c\}$ and $\{a, d\}$) and the extension $\{a, d\}$ is not an extension of the subframework $\{a, c, d\}$.

THEOREM 17. *nsa(SCOOC-naive) semantics violates Directionality, SCC-recursiveness and INRA.*

PROOF. A counterexample to Directionality and SCC-recursiveness is shown in Figure 3. A counterexample to INRA is shown in Figure 4.

THEOREM 18. *scc(SCOOC-naive) semantics violates INRA.*

PROOF. A counterexample is shown in Figure 5.

THEOREM 19. *SCF2 satisfies Weak Reinstatement, CF-Reinstatement, I-maximiality, Crash-resistance, Non-interference, Weak Directionality, Semi-directionality, Tightness, Conflift-Sensitiveness and Com-Closure.*

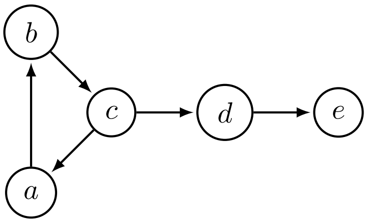

Figure 3. nsa(SCOOC-naive) semantics violates Directionality, because $\{b, e\}$ is an extension, even though $\{b\}$ is not an extension of the unattacked subframework $\{a, b, c, d\}$. nsa(SCOOC-naive) semantics violates SCC-recursiveness, because d is not in the extension $\{b, e\}$, even though the SCC $\{d\}$ is not attacked by an argument in this extension.

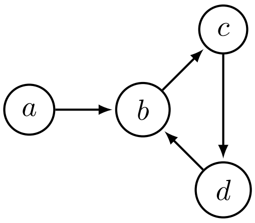

Figure 4. nsa(SCOOC-naive) semantics violates INRA, because b is attacked by every extension and the extension $\{a, d\}$ is not an extension of the subframework $\{a, c, d\}$.

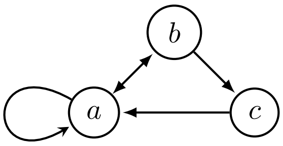

Figure 5. scc(SCOOC-naive) semantics violates INRA because a is attacked by every extension and the extension $\{c\}$ is not an extension of the subframework $\{b, c\}$.

PROOF. These results follow from the fact that SCF2 satisfies Naivety, Directionality and SCC-recursiveness using logical relations between principles shown by van der Torre and Vesic (2018).

Among the principles studied by van der Torre and Vesic (2018), there are also several ones that are not satisfied by SCF2:

THEOREM 20. *SCF2 does not satisfy any of the following principles: Defence, Admissibility, Indirect Conflict-Freeness, Reinstatement, Allowing Abstention, $\preceq_S^E$-Skepticism Adequacy and Succinctness.*

PROOF. A counterexample for Admissibility, Defence and Reinstatement is in Figure 6. A counterexample for Conflict-freeness is in Figure 7. A counterexample for Allowing Abstention is in Figure 8. A counterexample for $\preceq_S^E$-skepticism adequacy is in Figure 9. SCF2 does not satisfy Succinctness since SCF2 removes self-attacking arguments, so an attack between two self-attacking arguments is redundant. □

Note that apart from Succinctness, these principles are also not satisfied by CF2, stage and stage2.

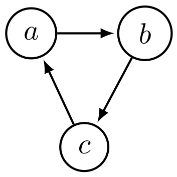

Figure 6. SCF2 violates admissibility, defence and reinstatement, since it returns three extensions: $\{a\}$, $\{b\}$ and $\{c\}$.

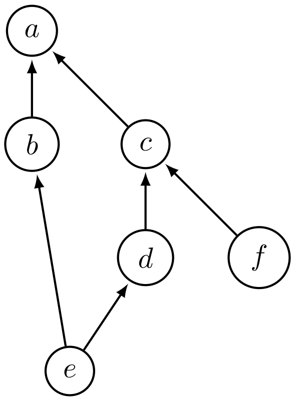

Figure 7. SCF2 violates indirect conflict-freeness. It yields an extension containing both a and e, even if e indirectly attacks a.

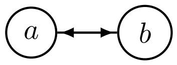

Figure 8. SCF2 violates Allowing Abstention, since $E_1 = \{a\}$ and $E_2 = \{b\}$ are extensions but there is no extension without a and b.

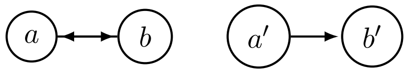

Figure 9. SCF2 violates $\preceq^E_S$-Skepticism Adequacy, since the Framework F on the left has two extensions $E_1 = \{a\}$ and $E_2 = \{b\}$, whereas the framework F' on the right has only one extension $E'_1 = \{a'\}$.

REFERENCES

Baroni, P. and Giacomin, M. (2007). On principle-based evaluation of extension-based argumentation semantics. *Artificial Intelligence* 171, 675–700. doi:10.1016/j.artint.2007.04.004. Argumentation in Artificial Intelligence

van der Torre, L. and Vesic, S. (2018). The principle-based approach to abstract argumentation semantics. In *Handbook of Formal Argumentation*, eds. P. Baroni, D. Gabbay, M. Giacomin, and L. van der Torre (College Publications)
